# Grade IV Glioma Potentially Disguised As COVID-19 Encephalitis

**DOI:** 10.7759/cureus.52075

**Published:** 2024-01-11

**Authors:** Austin Patrick Eisenberg, Nicolina Scibelli, Hannah Fischer, Victor Collier

**Affiliations:** 1 Internal Medicine, Grand Strand Medical Center, Myrtle Beach, USA; 2 Hematology and Oncology, University of Mississippi Medical Center, Jackson, USA; 3 Obstetrics and Gynaecology, Allegheny Health Network, Pittsburgh, USA

**Keywords:** acute cva, viral encephalitis, covid-19, glioblastoma multiforme, case report

## Abstract

Coronavirus disease 2019 (COVID-19) is caused by the severe acute respiratory distress syndrome coronavirus 2 (SARS-CoV-2), which became a pandemic in March 2020. Since that time, research has shed light on this disease's pulmonary, cardiac, and hematologic complications. However, we are still unraveling the complex neurologic sequelae of COVID-19. Here we present the case of a 58-year-old female who presented with weakness, gaze preference, and aphasia. She was diagnosed with a stroke which was managed medically. The patient returned two weeks later with memory loss and aphasia. An MRI was consistent with temporal lobe encephalitis, although a lumbar puncture was unremarkable. A polymerase chain reaction (PCR) test for COVID-19 was positive. Treatment was initiated for viral encephalitis with patient improvement. She was discharged a second time, and approximately three months later, she presented again with unrelenting headaches. Further imaging revealed a mass that was determined to be a grade IV glioma. Cases of glioma after viral encephalitis have been studied, but a clear link with COVID-19 has not been established.

## Introduction

Coronavirus disease 2019 (COVID-19) was declared a pandemic in March 2020 when the virus, severe acute respiratory distress syndrome coronavirus 2 (SARS-CoV-2), swept the globe. The classic symptoms of the disease are primarily respiratory, with a breadth of severity ranging from mild upper respiratory infection to acute respiratory distress syndrome (ARDS). Neurological presentations are less common and include stroke, seizures, encephalitis, encephalopathy, and aphasia [1-8]. Given its myriad of neurologic symptoms, it is important to recognize COVID-19 as a potential mimic of other neurologic diseases.

Encephalitis, an infection of the brain parenchyma, is commonly caused by viruses such as enteroviruses, herpesviruses, varicella zoster virus (VZV), and arboviruses [9]. Being predominantly a respiratory virus, COVID-19 can cause central nervous system (CNS) pathology due to its predilection for the respiratory tract. The virus gains access to host cells through the angiotensin‐converting enzyme 2 (ACE2) receptor, present in epithelial cells of the upper and lower respiratory tracts, amongst other cell types [9]. It is hypothesized that these viruses penetrate the CNS through the olfactory bulb after colonizing the epithelial cells of the upper respiratory tract [9, 10].

Glioma, a neoplasm of glial cells in the CNS, has been reported after or presenting as viral encephalitis [11-13]. Sayal et al. described the case of a patient with herpes simplex virus (HSV) encephalitis with imaging showing a right temporal lesion. The initial tissue biopsy was positive for HSV. The patient was successfully treated with acyclovir until a repeat presentation and subsequent tumor resection were positive for glioma [11]. Piper et al. presented three cases of patients who presented with encephalitis and had symptomatic improvement with acyclovir, despite negative cerebrospinal fluid (CSF) studies, who subsequently presented with gliomas [13].

Herein, we report a case of a woman with two distinct episodes of expressive aphasia followed by severe, unrelenting headaches who was originally diagnosed with a stroke. She presented two weeks after initial discharge and was subsequently diagnosed with temporal lobe encephalitis, possibly of COVID-19 origin. After a third presentation for unremitting headaches, she was ultimately diagnosed with grade IV glioma.

## Case presentation

**Figure 1:**
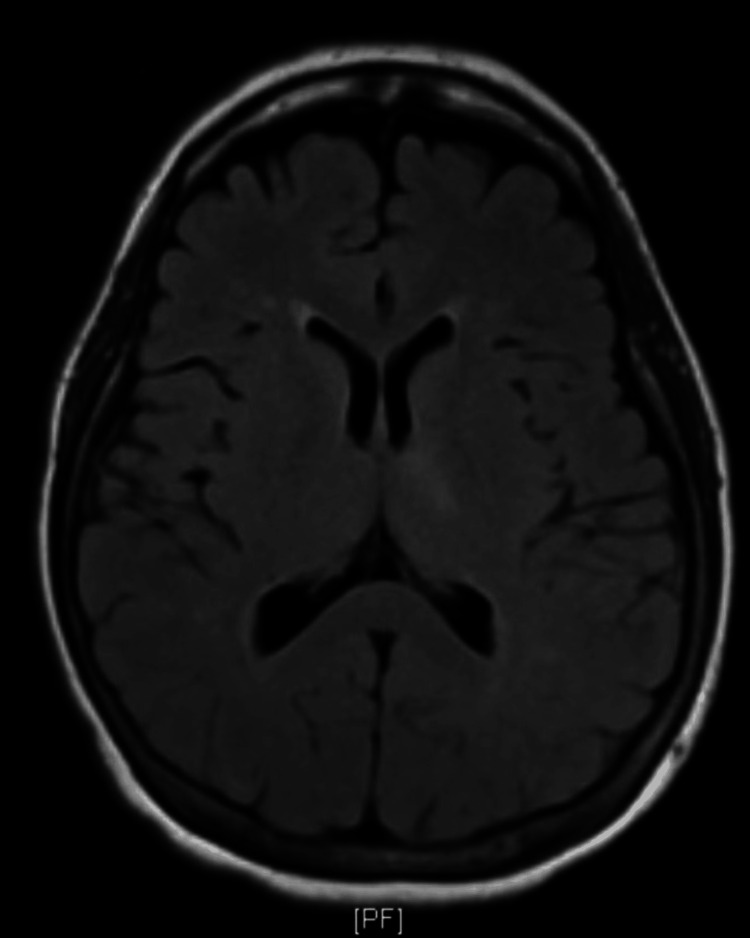
Axial plane T2 weighted MRI without contrast from the patient’s initial presentation: No radiographic abnormality was identified in this presentation.

A 58-year-old female with a medical history significant only for hypertension presented to the emergency department with a chief complaint of left-sided weakness, right gaze preference, and expressive aphasia. Her National Institutes of Health (NIH) Stroke Scale score was calculated at eight on arrival. On the neurologic exam, the patient was unable to answer questions regarding month and age, could not perform requested commands, displayed expressive aphasia, and was inattentive. The remainder of the exam was normal. A head CT without contrast revealed no acute intracranial pathology. A head-and-neck CT angiography (CTA) was performed, which did not show any large vessel occlusion or any flow-limiting carotid stenosis. Neurology was consulted, and tissue plasminogen activator (tPA) was administered. Brain MRI without contrast was negative for acute intracranial abnormalities (Figure 1).

The patient had a resolution of symptoms post-tPA and was discharged on atorvastatin, aspirin, and clopidogrel for a cerebrovascular accident (CVA).

**Figure 2:**
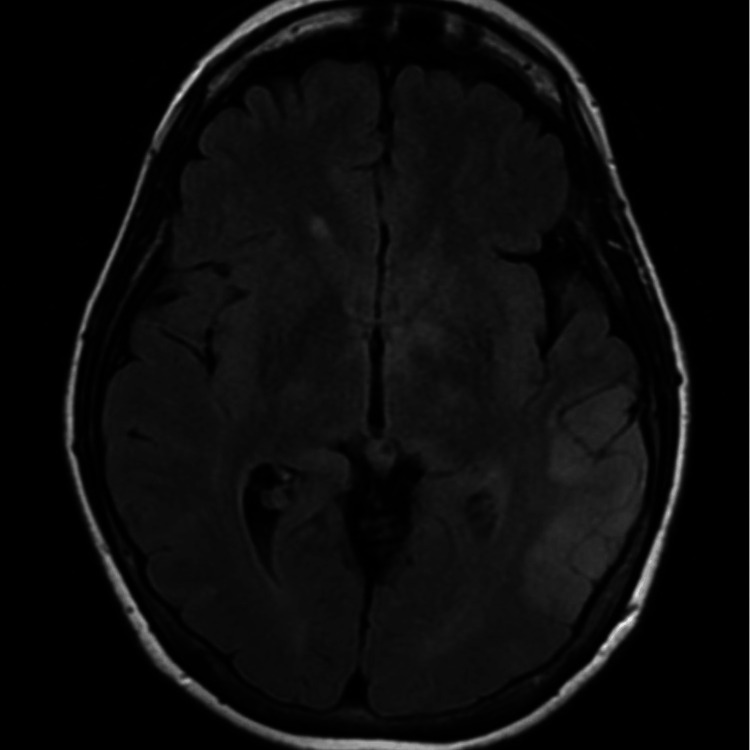
Axial plane T2 weighted MRI without contrast from the patient’s second presentation: The temporal lobe of this imaging shows an abnormal T2/FLAIR signal that was determined not to have imaging characteristics compatible with an infarct and was more consistent with encephalitis. FLAIR: fluid-attenuated inversion recovery

Approximately two weeks following the patient’s initial presentation, she returned to the emergency department with expressive aphasia, confusion, headache, and short-term memory loss. The patients’ NIH stroke scale score was four on admission. A head CT showed no evidence of an acute intracranial abnormality. A CTA of the head and neck showed no large vessel occlusion or flow-limiting cervical carotid stenosis. The brain MRI showed an abnormal T2/fluid-attenuated inversion recovery (FLAIR) signal involving the lateral posterior aspect of the left temporal lobe (Figure 2).

Findings were read to be highly suspicious for temporal lobe encephalitis, such as HSV encephalitis. A lumbar puncture was performed as well as an electroencephalogram (EEG). The CSF was clear and colorless, with three white blood cells per uL, two red blood cells per uL, glucose of 58 mg/dL, and elevated total protein of 70 g/L. Viral polymerase chain reaction (PCR) studies, including HSV-1, HSV-2, and VZV, were all negative. Gram stain and bacterial culture were also negative. The EEG showed periodic lateralized epileptiform discharges (PLEDs) in the left frontotemporal area. The neurologist interpreted that this EEG may lead to a decreased seizure threshold and was highly suspicious for HSV encephalitis. The patient was started on levetiracetam prophylactically. Given a recent COVID-19 exposure, the patient was tested with a nasopharyngeal swab for COVID-19 via real-time reverse transcriptase (RT)-PCR, which returned positive. The infectious disease specialist tendered the most likely diagnosis of COVID-19 encephalitis; however, the decision to administer empiric acyclovir was made due to MRI and EEG findings consistent with herpes encephalitis. The patient improved with intravenous acyclovir and was discharged with home acyclovir to be taken for a total of two weeks.

**Figure 3:**
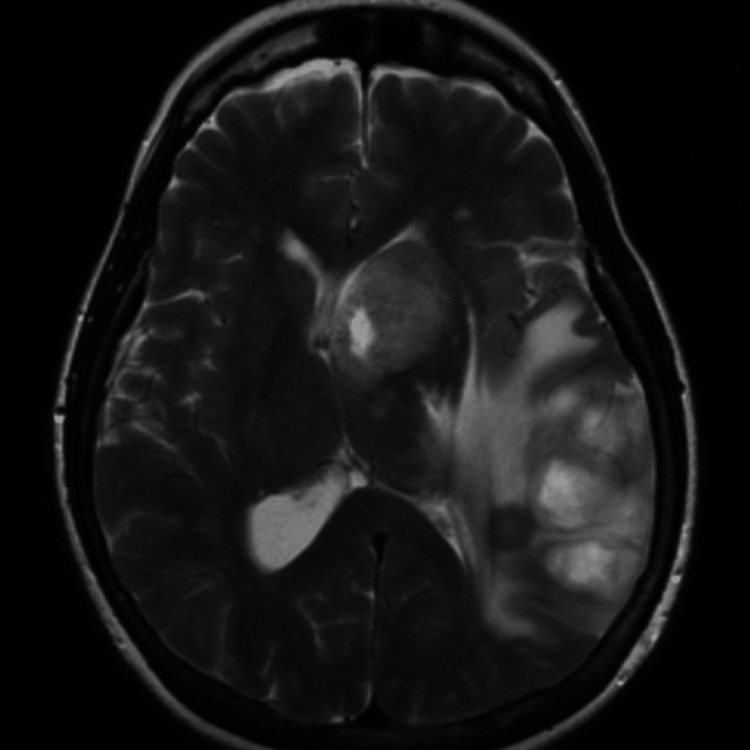
Axial plane T2 weighted MRI with and without contrast from the patient’s third presentation: Seen now in the temporal lobe is an irregular peripherally enhancing mass, measuring approximately 3.4 x 4.4 x 4.1cm, seemingly nodular and ill-defined in some areas with extensive surrounding edema. Also seen is a second peripherally enhancing mass centered in the left basal ganglia/caudate head, measuring approximately 2.9 x 2.7 x 2.6 cm. There is approximately 11 mm of left-to-right midline shift.

Three months following the patient’s original presentation, she presented to the outpatient clinic for evaluation of a new recurrence of headaches. During this appointment, she stated that her headaches had shown improvement for about a month but returned and were unbearable for the past two weeks. The patient was otherwise hemodynamically stable, and MRIs with and without contrast of her brain were ordered. The MRI showed an irregular and nodular peripherally enhancing mass, measuring approximately 3.4 x 4.4 x 4.1 cm, with extensive surrounding edema (Figure 3).

There was also a second peripherally enhanced mass in the left basal ganglia, measuring approximately 2.9 x 2.7 x 2.6 cm, with possible extension across the midline leading to a left-to-right midline shift. The patient was instructed to return to the ED and was admitted to be examined and treated by neurosurgery. Neurosurgery performed a stereotactic needle biopsy of the left parietal lobe mass, which returned with pathology consistent with grade IV glioma. The patient was not a surgical candidate and underwent cone-beam CT-guided radiation with concurrent chemotherapy (temozolomide). However, she had progressed with her disease, was ultimately transitioned to hospice care, and died at home with her family.

## Discussion

Typical features seen in COVID-19 encephalitis are confusion, agitation, delirium, and coma [8]. Our patient experienced some symptoms of confusion and memory impairment, but her most predominant symptom was expressive aphasia. The MRI revealed a T2/FLAIR signal in the left temporal lobe. Studies have shown that neuroimaging in COVID-19 encephalitis is typically a cortical or subcortical white matter T2/FLAIR signal hyperintensity [14]. The pathogenesis behind the CNS invasion is through two proposed mechanisms. The first is that the SARS-CoV-2 virus spike glycoprotein binds to the ACE2 receptor, facilitating entrance to the brain. The second proposed mechanism is that the virus enters the brain through the olfactory system. This viral entry eventually leads to an inflammatory response against the virus, triggering a cytokine storm, with hypoxic and metabolic insults subsequently resulting in diffuse brain dysfunction [7]. Given the unknown connection between glioma and viral encephalitis, it is possible that the SARS-CoV-2 virus was also associated with our patient's illness.

Herpes simplex virus encephalitis and glioma have often been linked in the literature. Sayal et al. chronicled the case of a patient with concurrent HSV encephalitis and glioma, with imaging showing a right temporal enhancing lesion with mass effect. The initial histology of the resected tumor was positive for HSV. The patient was successfully treated with acyclovir until repeat presentation and tumor resection was positive for glioma [11]. It is unclear if our patient had COVID-19 encephalitis or if her symptoms were secondary to progressive glioma. Imaging on the second presentation was consistent with encephalitis, but this does not rule out the possibility of glioblastoma mimicking viral encephalitis.

A study by Gregory et al. showed that patients with previously diagnosed gliomas had significant disease progression after COVID-19 infection [15]. Glioma tumor cells have increased expression of endothelial growth factor receptors and vascular endothelial growth factor receptors, amongst many other receptors that increase tumorigenesis and tissue invasion [16]. Khan et al. showed that the spike protein on COVID-19 has increased affinity for vascular endothelial growth factor receptor and endothelial growth factor receptor, which may explain the rapid glioma progression after COVID-19 infection reported in the study by Gregory et al. [15, 17].

The imaging from the cases presented by Rees et al. showed high signal abnormalities in the temporal lobes, similar to the MRI in our patient and consistent with the typical presentation of HSV encephalitis [12]. To add to the difficulty of our diagnosis, antiviral administration has been shown to relieve symptoms in cases of encephalitis that were ultimately diagnosed as gliomas. As described by Piper et al., three patients believed to have HSV encephalitis were started on IV acyclovir and had improved neurological function. After cessation of acyclovir, all of the patients had a relapse of symptoms and a final diagnosis of grade IV glioma [13]. Our patient also experienced improvement following acyclovir administration, confounding the clinical picture. In light of all the presented data, it is unclear whether our patient developed a glioma secondary to COVID-19 encephalitis or had an undiagnosed glioma that rapidly progressed after COVID-19 encephalitis. This case demonstrates the need for further research on the relationship between COVID-19 and the subsequent development of CNS malignancies such as gliomas and the pathophysiology behind rapid glioma progression after COVID-19 infection.

## Conclusions

Several cases connecting glioma to prior viral encephalitis have been reported, though no direct causality has been established. Encephalitis secondary to COVID-19 infection is now a known complication of the virus, with several case reports having documented this phenomenon. Our patient experienced two separate symptomatic periods with complete improvement of symptoms in between, followed by a new development of unbearable headaches and a final diagnosis of glioma. It has been shown that COVID-19 infection increases glioma progression, but it is unclear whether viral encephalitis, caused by COVID-19 or otherwise, may be directly linked to new glioma development. We propose that this phenomenon be further studied to determine whether a relationship exists between the two conditions. If an association exists, patients with presumed viral encephalitis with negative serologic and CSF studies should always have glioblastoma multiforme in the differential diagnosis.
